# The ovulation trigger method affects gonadotropin concentrations and gonadotropin receptor expression during final oocyte maturation in women

**DOI:** 10.3389/fendo.2026.1791342

**Published:** 2026-03-16

**Authors:** Liv La Cour Poulsen, Malene Louise Johannsen, Marie Louise Grøndahl, Marie Louise Wissing, Claus Yding Andersen

**Affiliations:** 1 The Fertility Clinic, Department of Gynecology & Obstetrics, Copenhagen University Hospital Herlev, Herlev, Denmark; 2 Toxicology and Drug Metabolism, Department of Pharmacy, Faculty of Health and Medical Sciences, University of Copenhagen, Copenhagen, Denmark; 3 Alleris Fertility, Søborg, Denmark; 4 Institute of Clinical Medicine, Faculty of Health and Medical Sciences, University of Copenhagen, Copenhagen, Denmark; 5 Department of Urology, Copenhagen University Hospital - Herlev and Gentofte Hospital, Herlev, Denmark

**Keywords:** FSH receptor, hCG, LH receptor, oocyte maturation, ovarian stimulation, ovulation, ovulation trigger

## Abstract

**Introduction:**

Human chorionic gonadotropin (hCG) and gonadotropin-releasing hormone agonist (GnRHa) are widely used for final maturation of follicles in fertility treatment, yet the detailed dynamics of follicle stimulating hormone (FSH), luteinizing hormone (LH)/hCG and their receptor expression (i.e. FSHR and LHR) during the ovulatory process remain insufficiently characterized.

**Material and methods:**

This prospective, single-center study included 50 women undergoing ovarian stimulation during 2016-2018. Each participant contributed one follicle aspirated at T = 0,12,17 or 32h after trigger administration (0.5 mg GnRHa (buserelin) or 6,500 IU hCG) and a second follicle aspirated at oocyte pickup (T = 36h). Follicular fluid (FF) and plasma were analyzed for gonadotropins, and granulosa cells for *FSHR* and *LHR* transcript levels.

**Results:**

GnRHa triggered a rapid endogenous surge in plasma: LH peaked at ≈120–140 IU/L at 12h and remained elevated until 36h; FSH rose from ≈17 to ≈25 IU/L within 12h. In FF, LH exceeded 100 IU/L at 17 h, whereas FSH remained low with a small peak at 12h (8–9 IU/L). With hCG triggering, circulating FSH declined to ≈40% of baseline by 17h, while FF FSH was constantly low (≈5 IU/L) and unchanged. FF hCG concentrations increased sharply between 17–32h. Consequently, LH appeared earlier (12–17h) in FF than hCG (17-32h). *LHR* granulosa cell expression decreased to ≈3-5% of baseline by 32h, indicating substantial downregulation as ovulation progressed. *FSHR* was downregulated even faster, between 0-12h.

**Discussion:**

GnRHa induces a stronger and more physiologic gonadotropin surge than commonly assumed, with timely entry of LH and FSH into FF coinciding with high *FSHR* and *LHR* expression and with the known timing of intrafollicular oocyte-maturation signals (12–17h). In contrast, hCG enters the follicle later, when *LHR* expression is markedly reduced, and does not provide an FSH component. This may explain reports of slightly higher MII rates using the GnRHa trigger. These findings highlight the importance of temporal receptor dynamics in optimizing final oocyte maturation and support the use of GnRHa as a stand-alone trigger in freeze-all antagonist cycles and in combination with hCG (i.e., dual triggering) in fresh cycles to optimize oocyte maturation.

## Introduction

The rationale for ovarian stimulation (OS) with exogenous gonadotropins is to increase the number of preovulatory follicles and retrieve multiple mature oocytes for use in assisted reproductive technologies (ART). Ovarian stimulation protocols have, over the past five decades, evolved from simple oral ovulation induction to highly individualized, evidence-based regimens aimed at optimizing both efficacy and safety.

A variety of OS protocols have been developed to regulate follicular recruitment and maturation. The earliest included clomiphene citrate, which increases pituitary gonadotropin release by antagonizing hypothalamic estrogen feedback. Subsequently, the introduction of gonadotropin-releasing hormone agonist (GnRHa) protocols enabled pituitary downregulation and prevention of premature luteinizing hormone (LH) surges. The GnRHa regimens were later complemented by the GnRH antagonist protocol, which allows rapid, reversible pituitary suppression and has become the most widely used approach in modern ART.

Alongside these protocols, a range of gonadotropin preparations have been introduced, including urinary-derived human menopausal gonadotropins (hMG), highly purified follicle stimulation hormone (FSH), and recombinant FSH and LH produced in Chinese hamster ovary or human cell lines (1). These advances have allowed for more standardized, predictable, and customizable OS strategies tailored to the patient’s endocrine profile and ovarian response (1).

A key step in all OS protocols is the induction of final follicular maturation, which aims at mimicking the natural mid-cycle gonadotropin surge. Traditionally, this is achieved by administration of human chorionic gonadotropin (hCG), which acts as an LH receptor agonist with a prolonged half-life. More recently, the use of GnRHa as an “ovulation trigger” in GnRH antagonist cycles gained popularity (2–4), as it induces a physiological, albeit shorter-lived, pituitary surge of both LH and FSH (5, 6). While hCG provides sustained LH-like activity, it lacks an FSH component, and the GnRHa-induced surge—although more physiologic—is typically blunted compared to the natural cycle (5, 6).

The ovulatory process itself is a remarkably dynamic and tightly coordinated sequence of events, encompassing oocyte resumption of meiosis, cumulus expansion, follicular rupture, luteinization of theca – and granulosa cells (GCs), and the initiation of progesterone secretion (7, 8). These transformations occur within a narrow window of approximately 36 hours. The interplay between LH and FSH surges is essential, not only for oocyte maturation and release but also for establishing a functional corpus luteum capable of supporting early luteal phase steroidogenesis (9, 10).

Despite decades of clinical use, surprisingly little is known about the detailed dynamics of gonadotropins in both circulation and follicular fluid (FF) during the periovulatory period and their receptor expression in GCs. In particular, the relative contribution of LH-like and FSH-like activity to follicular processes such as steroidogenesis, cumulus expansion, and luteinization remains incompletely understood. Furthermore, the expression of gonadotropin receptors (i.e., LHR and FSHR) on GCs—and their regulation during this brief, but critical window—has not been systematically characterized *in vivo* in human follicles.

The present study aims to provide a comprehensive description of the temporal course of circulation and follicular concentrations of gonadotropins, and the expression of *FSHR* and *LHR* in GCs, following administration of either hCG or GnRHa as the ovulation trigger

By comparing these two clinically established trigger approaches, this study seeks to elucidate key differences in gonadotropin action and receptor dynamics in the periovulatory period, which may provide important insights for optimizing OS protocols, improving oocyte competence, and refining luteal phase support strategies in ART.

## Materials and methods

### Ethical approval

The study was approved by The Scientific Ethical Committee of Region Zealand, Denmark (SJ-530), and the Danish Data Protection Agency (8).

### Participants

The study was conducted as a prospective cohort at the University Hospital-affiliated Fertility Clinic, Holbæk Hospital, Region Zealand, Denmark, during September 2016 to March 2018, as previously described (7, 8). A total of 50 women were included. Included women were below 36 years of age, undergoing IVF or ICSI treatment because of infertility due to male factor, tubal factor, unexplained infertility, or non-hyperandrogenic PCOS (women with PCOS due to oligo/anovulation and polycystic ovaries could be included, while women with elevated s-androgens were excluded). For ethical reasons, only women who had developed at least nine mature follicles ≥ 14 mm at the last control ultrasound before ovulation trigger were asked to participate. The women were treated with a standard antagonist protocol initiated at cycle day 2 or 3 with individually dosed recombinant FSH (n = 42, Puregon^®^, MSD, Denmark) or hMG (n = 8, Menopur^®^, Ferring, Denmark) and a GnRH antagonist (Ganirelix 0.25 mg, Fyremadel^®^, SUN pharma, Netherlands) from stimulation day 5. To avoid ovarian hyperstimulation syndrome (OHSS), ovulation was induced with either recombinant hCG (rhCG, 6500 IU, Ovitrelle^®^, Merck, Germany) or GnRHa (Buserelin 0.5 mg, Suprefact, Sanofi-Aventis, France) according to the number of ovarian follicles at the final control visit, as evaluated by ultrasound and/or early clinical signs of OHSS. Oocyte pick up (OPU) was performed 36 hours after ovulation trigger.

The participants donated FF and GCs from one mature follicle (≥14mm) collected by transvaginal ultrasound guided follicle puncture and aspiration. Twenty-three women donated one follicle at T = 0h (before ovulation trigger), 10 women at T = 12h, 6 women at T = 17h, and 11 women at T = 32h. All 50 women had FF and GCs collected at oocyte pick-up (OPU) from the first aspirated follicle containing an oocyte. Thus, each participant contributed two samples in total. The specific time points (T = 0h, 12h, 17h and 32h) were chosen to cover the 36h peri-ovulatory period, while convenience to the patients and the fertility clinic was taken into consideration. Patients were allowed to choose which 0h-32h aspiration they preferred. OPU was performed at T = 36h as this was standard practice in the Fertility Clinic. Previous reports have described the hormonal and growth factor profiles, as well as relevant patient characteristics, for this cohort (7, 10, 11).

Among the 23 women donating follicles at T = 0h, 15 women underwent OS with rFSH (Puregon^®^) and 8 with hMG (Menopur^®^).

### Baseline descriptive parameters

Baseline descriptive parameters included age, BMI, cause of infertility, and evaluation of serum LH, FSH, estradiol, AMH, prolactin and thyroid stimulating hormone (TSH) on cycle day 2-3. The serum hormone analyses were performed as routine analyses at the hospital’s biochemical laboratory. At the final control ultrasound before ovulation trigger, the number of mature follicles ≥ 14 mm was counted.

### Isolation of granulosa cells and follicular fluid

Collection of GCs and FF were also previously described (8). In short, GCs were isolated from the aspirated FF with a 100 µL pipette through a light microscope and subsequently washed through a 4-well dish containing phosphate buffered saline (PBS) and 0.1% polyvinyl alcohol (PVA) (Sigma-Aldrich, Denmark), transferred to a 0.2 mL cryotube (MicroAmp, Applied Biosystems, CA, USA) and snap-frozen in liquid nitrogen.

The FF was centrifuged at 300 g for 10 min to remove cell debris and the supernatant was collected and stored in cryovials (1.5 ml, NUNC, Fisher Scientific, Denmark) at – 80 °C until further analyses. All samples were macroscopically clear or contained only trace amounts of blood.

### Plasma samples

Within 30 min before the follicle puncture and OPU, a blood sample was collected from each woman. The sample was collected in an EDTA tube, centrifuged at 2000 g for 20 min, and the supernatant was collected and stored in cryovials (1.5 ml, NUNC, Fisher Scientific, Denmark) at –80 °C until further analyses.

### Enzyme-linked immunosorbent assays

In FF and plasma, concentrations of FSH, LH and hCG were measured using commercially available ELISA kits: FSH (RE52121, IBL, Triolab, Brøndby, Denmark), LH (DNOV030, Novatec Immundiagnostica GmbH, Dietzenbach, Germany), and hCG (RAB0092, Sigma Aldrich, St. Louis, MO, USA), according to the manufacturers’ instructions. The coefficients of variation (CV) were for LH: intra-assay < 9.21%; inter-assay < 7.91, FSH: intra-assay < 7.91%; inter-assay < 7.18%, hCG: intra-assay <10%; inter-assay < 12%. LH and FSH were measured in all samples at all time points, however, LH were below the detection limit of the kit in almost all samples at T = 0h. HCG were only measured in rhCG triggered patients at T = 12h-36h.

Concentrations of the peptide hormones AMH, inhibin A, and inhibin B were measured in FF at 0h and 36h using special ELISA kits (AL-105-i, AL-123-i and AL-107-I, respectively, Ansh Labs, TX, USA). All ELISAs were performed according to the manufacturers’ instructions. The CV were for AMH: intra-assay < 4.0%; inter-assay < 4.8%, inhibin A: intra-assay < 4.3%; inter-assay < 5.3%; inhibin B: intra-assay < 6.3%; inter-assay < 4.0%.

Prior to analysis, FF or plasma was diluted in PBS with 1% bovine serum albumin as appropriately to fit the detection range of the kit. Samples with concentrations below the lower detection limit were assigned the value of the lower detection limit (16/18 for LH at 0h, while the remaining had 100% valid values.

### Mass spectrometry analysis of steroids

Sex steroid hormones in FF were quantitated at T = 0h and T = 36h using a previously published protocol (Johanssen et al., 2025). In brief, steroids were extracted using solid-phase extraction and quantified by liquid chromatography/mass spectrometry, using a method fully validated and developed by Weisser et al. (12). This method adheres to the International Council for Harmonisation (ICH) guidelines (13) and enables the simultaneous quantification of 20 steroids involved in steroidogenesis.

### Statistical analyses

All tests described in this section were performed with SPSS (v25, IBM, NY, USA). Unless otherwise mentioned, *P <*0.05 was considered significant.

Continuous baseline descriptive parameters between time points were compared with a Kruskal–Wallis test followed by *post hoc* test with a Bonferroni correction as a normal distribution could not be assumed and because study groups were of unequal size. Continuous baseline parameters between trigger groups were similarly compared with a Mann Whitney U test. Categorical parameters were compared by Fisher’s exact test.

Differences in gonadotropin concentration across time points were tested in a mixed model with ‘time’ and ‘trigger drug’ as fixed factors and ‘patient-ID’ as a random factor, which takes into account the pairing of samples. The model used the first order autoregressive (AR(1)) covariance matrix as this resulted in the best model fit. Differences between women treated with hMG and rFSH at T = 0h and T = 36h were tested with an independent t-test. Prior to statistical modelling, all FF and plasma gonadotropin concentrations were log-transformed to ensure a normal distribution.

### LH and FSH receptor expression in granulosa cells

*LHR* and *FSHR* expression was drawn from a microarray analysis, which has been previously published (8). RNA isolation and details of the microarray analysis were described thoroughly in that paper. In short, the microarray platform was the Clariom D™ Pico Assay (Applied Biosystems, Thermo Fisher, MA, USA), and the data was analyzed using the Transcriptome Analysis Console (TAC 4.0.1, Thermo Fisher Scientific, MA, USA). 83 CEL-files were of sufficient quality, representing time points T = 0h (n = 17), T = 12h (n = 7), T = 17h (n = 6), T = 32h (n = 9) and T = 36h (n = 44). The differential expression analysis was setup using ANOVA ebayes comparisons with an advanced random factor for ‘patient ID’, accounting for the pairing of samples. Differential expression with a false discovery rate (FDR) < 0.01 combined with a gene expression fold change > 2 was considered significant. Raw and processed microarray data were deposited to the Gene Expression Omnibus (http://www.ncbi.nlm.nih.gov/geo, accession number: GSE133868).

Expression of leukocyte specific marker *PTPRC* (CD45) was consistently very low across time points indicating no or low leukocyte contamination with no differences between time points.

For the present study, only *LHR* and *FSHR* expression were evaluated to support and discuss the role of gonadotropins during ovulation.

## Results

The concentrations of all three gonadotropins were significantly regulated across time points (p < 0.001), except for the FSH concentration in follicular fluid. There was a significant effect of trigger modality for LH and FSH in serum and LH in FF (p < 0.05) while this effect was only near-significant for FSH in FF (p = 0.065).

**Figure 1:**
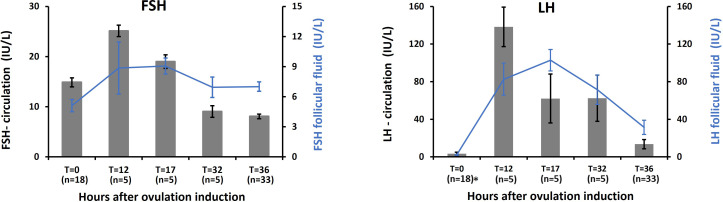
GnRH agonist trigger: Levels of FSH and LH in circulation and in follicles of 33 women receiving GnRH agonist trigger for final maturation of follicles following ovarian stimulation. *At T = 0h, LH concentrations were below the assay detection limit in 16 paired plasma and follicular fluid samples.

The temporal profiles of circulating LH and FSH following a GnRHa trigger in 33 women are shown in **Figure 1**. LH concentrations peaked at ≈ 130 IU/L at T = 12h, while FSH increased from ≈17 IU/L to ≈25 IU/L at the same time point. Both gonadotropins declined thereafter as OPU approached.

In FF, LH concentrations were close to 100 IU/L already at T = 12h and peaked at T = 17h, where it exceeded 100 IU/L, while FF FSH concentrations increased only slightly at T = 12-17h, with a range of 6 to 9 IU/L.

**Figure 2:**
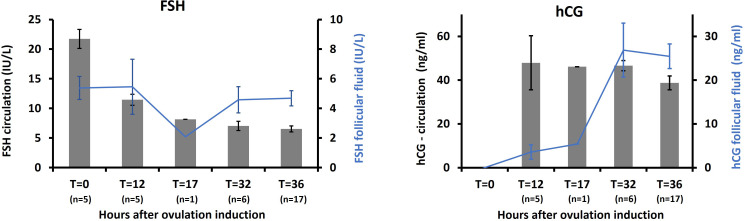
hCG-trigger: Levels of FSH and hCG in circulation and in follicles in 17 women receiving hCG trigger for final maturation of follicles following ovarian stimulation.

In 17 women receiving hCG for final maturation of follicles, the FSH concentration in plasma declined throughout the periovulatory period, while the FF FSH concentration remained relatively constant (**Figure 2**). Concentrations of hCG in plasma rose quickly to a relatively constant high level from T = 12h and onwards, while FF hCG levels were low until a sharp increase at T = 32h and 36h.

**Figure 3:**
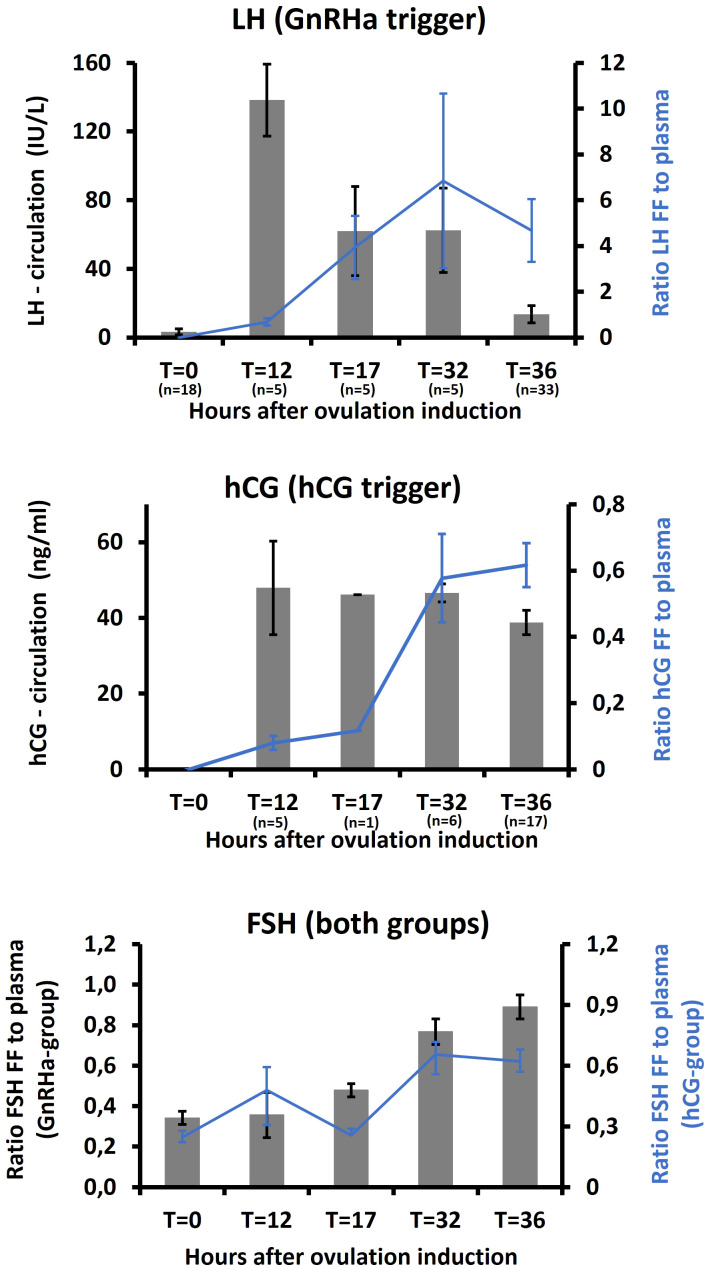
Levels of LH and hCG in circulation and the ratio of LH and hCG in follicular fluid and circulation in women receiving GnRH agonist and hCG trigger, respectively, for final maturation of follicles following ovarian stimulation.

The ratio of FF to plasma concentrations of the gonadotropins are depicted in **Figure 3** and show that the permeability of the follicle is affected during final maturation of follicles with increasing FF to plasma ratios overall. It seems that in the GnRHa group, there is a slightly faster follicle entry of both LH and FSH, while hCG only increased at T = 32h in the hCG group. In addition, the FF to plasma ratio of FSH appears to be higher in the GnRHa group.

**Figure 4:**
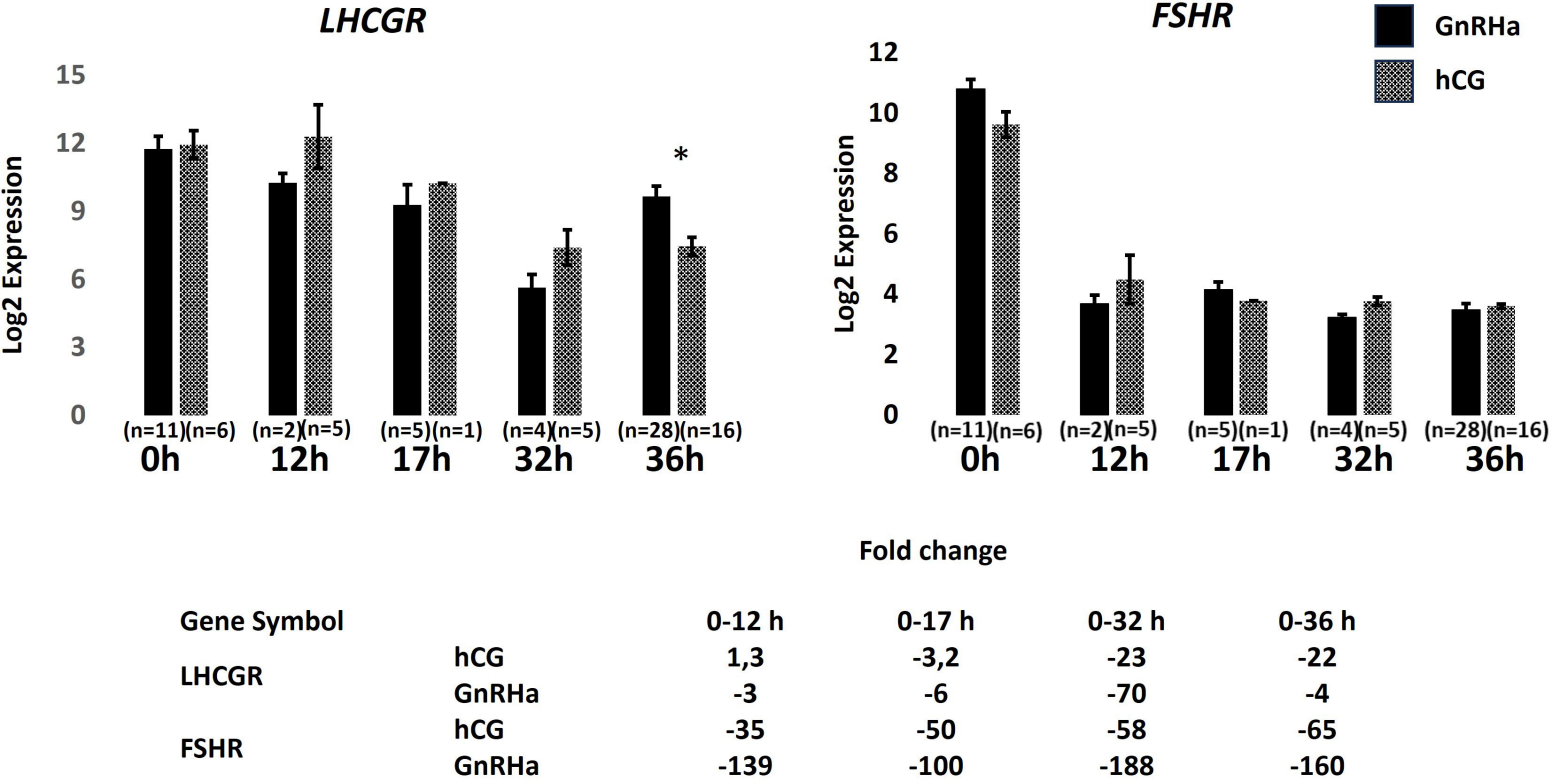
Gonadotropin receptor expression during the mid-cycle surge of gonadotropins in women undergoing ovarian stimulation receiving final maturation of follicles with either a GnRH agonist or hCG trigger. Error bars = ± SEM. *Significant difference, FDR < 0,01.

Gene expression data for *FSHR* and *LHR* in GCs across the different time points in relation to whether the woman received hCG trigger or GnRHa trigger is shown in **Figure 4**. Overall, the temporal downregulation of receptor expression is similar between the two modes of ovulation induction. Down-regulation of *FSHR* expression occurred fast being only a few percent of starting levels at T = 12h. The gene expression of *LHR* was also strongly downregulated, but later compared to *FSHR*, at T = 17-32h. In addition, it is noticeable that the *LHR* expression recovered significantly faster in GnRHa trigger group compared to the hCG group at T = 36h.

Spearman correlation analysis of FSH concentration at T = 0h versus either *FSHR* or *LHR* expression at T = 0h did not show any significant associations (p>0.10) and there was no significant association between *FSHR* and *LHR* expression at T = 0h. In addition, gene expression of *FSHR* and *LHR* in GCs were similar at T = 0h irrespective of whether the woman received hMG or rFSH for OS (data not shown).

Of the 23 women donating follicles at T = 0h, 8 received hMG for ovarian stimulation, and 15 received rFSH. Steroid and growth factor concentrations at T = 0h and T = 36h are summarized in **Table 1**. At T = 0h significantly increased concentrations of testosterone and borderline increased concentrations of estradiol were found in FF from women receiving hMG, and concentrations of FSH in FF were also increased. The difference in testosterone concentrations persisted at T = 36h, while concentrations of estradiol and FF-FSH were similar between groups. Conversely, progesterone concentrations were significantly lower in FF from the hMG group at T = 36h. All other hormonal parameters remained similar between the two types of gonadotropin stimulation. Baseline parameters such as age, BMI, total dose of FSH administered, and volume of the aspirated follicle were similar between women treated with hMG and rFSH.

**Table 1:** Concentrations of steroids and growth factors in follicular fluid collected at the time of ovulation induction (T = 0h) and at the time of OPU (T = 36h) from the same woman in relation to the gonadotropin preparation used for ovarian stimulation.

Steroid (unit)	T = 0h			T = 36h		
	hMG (N = 8)	rFSH (N = 15)	P value	hMG (N = 8)	rFSH (N = 15)	P value
FSH plasma (IU/L)	14.9 ± 1.3	17.9 ± 1.1	P=0.057	7.5 ± 1.0	7.9 ± 1.2	NS
	16.1 [9.1,19.4]	18.3 [9.8,26.2]		7.1 [3.7,12.8]	6.3 [3.4,23.4]	
FSH follicular fluid (IU/L)	6.4 ± 0.7	4.5 ± 0.4	P<0.02	6.8 ± 1.1	5.5 ± 0.7	NS
	5.8 [4.1,10.2]	4.3 [1.3,8.4]		6.5 [1.6,12.0]	6.3 [1.1,10,3]	
Progesterone (µmol/L)	4.1 ± 1.2	4.9 ± 0.7*	NS	11.0 ± 1.0	20.5 ± 3.0	P =0.017
	3.3 [0.6,12.3]	5.6 [1.4,7.2]		11.0 [7.2,15.3]	23,2 [3.6, 38.8]	
17OH-Progesterone(µmol/L)	5.3 ± 1.8	5.1 ± 1.1	NS	5.0 ± 0.6	4.8 ± 0.5	NS
	3.2 [1.1, 15.6]	4.4 [1.4,12.1]		4.9 [2.9,8.6]	4.8 [2.3,9,3]	
Androstenedione (nmol/L)	6.7 ± 1.5	6.0 ± 2.0	NS	6.2 ± 1.8	3.7 ± 0.9	NS
	4.9 [2.9,11.3]	3.9 [0.6,18,7]		6.9 [0.6,14.2]	2.1 [0.6,11,3]	
Testosterone (nmol/L)	1.5 ± 0.17	1.1 ± 0.10	P=0.032	1.8 ± 0.2	1.2 ± 0-14	P =0.038
	1.3 [1.1,2,3]	1.2 [0.6,1.7]		1.9 [1.1,2.5]	1.2 [0.8,2.5]	
Estradiol (µmol/L)	9.4 ± 1.8	5.8 ± 1.2	P=0.060	1.3 ± 0.3	1.2 ± 0.2	NS
	9.0 [2.4,16.1]	5.1 [1.0,12.3]		1.1 [0.3,2.5]	1.0 [0.1,2.7]	
AMH (ng/ml)	23.6 ± 12.6	6.6 ± 1.7	NS	4.5 ± 0.9	3.3 ± 0.5	NS
	6.5 [4.2,116]	3.9 [0.1,27.5]		5.2 [1.1,7.8]	2.9 [1.2,7.5]	
Inhibin-B (ng/ml)	551 ± 105	352 ± 75*	NS	125 ± 33	83 ± 12	NS
	532 [126,1.084]	254 [89,872		102 [36,354]	75 [29,198]	
Inhibin-A (ng/ml)	127 ± 14	124 ± 9	NS	97 ± 14	108 ± 11	NS
	117[81,213]	119 [74,167]		106 [31, 154]	96 [41,190]	

Mean [± SEM] and Median [range]. Steroids measurements by LC-MS/MS (Johannsen et al., 2025), other hormones measured by ELISA. NS: Not significant. *One outlier of 1.865 ng/ml were excluded.

Baseline parameters between ovulation trigger groups showed expected differences in number of follicles at final control visit (p = 0.001) and AMH (p = 0.033), as well as a difference in BMI (median 20.3 (hCG) versus 23.5 (GnRHa), p = 0.0001) and baseline FSH level at cycle day 2-3 (6.7 (hCG) versus 5.3 (GnRHa, p = 0.014), which is unlikely to affect the peri-ovulatory gonadotropin concentrations and receptor-expression. The distribution of stimulation drug was uneven with 8/33 women in the GnRHa group stimulated with hMG, while all 17 women received rFSH in the rhCG group.

We do not have information on the total number of oocytes or total number of metaphase II oocytes in each trigger group, but the first aspirated oocyte at OPU was metaphase II in 30/33 women triggered with GnRHa, and 15/17 women triggered with rhCG (p > 0.05).

## Discussion

This study provides new insight into the hormonal environment induced by GnRHa and hCG triggers during the process of final maturation of follicles. Following a GnRHa trigger, a robust endogenous gonadotropin surge in women undergoing OS occurred. After the trigger, LH rose rapidly to a peak of ≈130 IU/L at T = 12h and remained on average elevated at around 60 IU/L at T = 17h and T = 32h before becoming reduced at T = 36h. FSH levels were already within the natural cycle ovulatory range at T = 0h (≈10–14 IU/L) and increased further to ≈25 IU/L during the first 12h. These findings challenge the common clinical assumption that the GnRHa-induced surge is weaker than its physiological counterpart (5, 6, 14, 15). Instead, these data show that both FSH and LH reach substantially higher peak concentrations and display a comparable duration (>24 hours) as compared to the physiological surge. Collectively, this means that a standard antagonist protocol followed by a GnRHa trigger reliably generates an ovulatory stimulus supporting oocyte maturation.

In women receiving an hCG trigger, plasma FSH declined rapidly from ≈20–22 IU/L at T = 0h to ≈40% of baseline by T = 17h and continued to decrease until oocyte retrieval. These results confirm that hCG-trigger protocols provide no FSH support during the periovulatory interval and rely solely on LH receptor activation by hCG. Meanwhile, circulating hCG levels rose sharply from T = 0h to T = 12h and remained high until OPU, providing a strong and persistent signal for final follicular maturation and subsequent luteal support and confirming the robustness of this long-standing clinical standard.

FSH concentrations in FF after the GnRHa trigger were significantly higher than those measured in FF from women receiving hCG and rose to peak at T = 12-17h, demonstrating a clear intrafollicular consequence of the endogenous surge. Nonetheless, absolute FSH values remained in the single-digit range throughout the periovulatory interval for both trigger modalities. In contrast, FF LH concentrations in the GnRHa group were markedly elevated already at 12-17h and exceeded corresponding plasma levels at T = 17h, T = 32h, and T = 36h, suggesting preferential accumulation of LH within the follicular compartment and providing a strong luteinizing signal to the GCs. In the hCG-trigger group, levels of FF hCG did not reach the levels of LH in the GnRHa group until T = 32h. The temporal differences between LH entry into FF following GnRHa and hCG entry following an exogenous hCG trigger are particularly noteworthy. Previous studies indicate that the signals within FF that drive oocyte maturation and progression to the MII stage peaks shortly after ovulation induction within the first 12–17h of the ovulatory process (9). Several reports also show slightly but consistently higher MII rates after GnRHa trigger compared with hCG triggers (16–19) and there is some indication that the concept of dual trigger (i.e. simultaneous injection of hCG and GnRHa) improves the oocyte numbers and maturation rate compared to hCG alone (20). The present findings may provide a physiological explanation for this observation: LH enters the follicle earlier than hCG, thereby initiating the maturation cascade at an earlier and more physiologically appropriate time point. This interpretation is further supported by the observed FF/plasma ratios: in the GnRHa group, the LH FF/plasma ratio rose already at T = 12h, whereas an increase in the hCG FF/plasma ratio was only evident from T = 32h and will therefore take place sometime between T = 17h and T = 32h.

These temporal dynamics of gonadotropin entry into the follicle may reflect the permeability of the basal membrane surrounding the follicles (21, 22) and it should be considered alongside the changing expression of gonadotropin receptors in GCs. *LHR* expression decreased progressively and reached a nadir at T = 32h, with levels reaching only ≈3-5% of those measured at T = 0h. The rise of hCG within the follicle in the hCG-trigger group therefore occurs at a time when *LHR* expression is considerably reduced. In contrast, the rapid entry of LH into FF after a GnRHa trigger coincides with much higher *LHR* expression levels during the first 12–17h. This alignment between ligand availability and receptor abundance likely optimizes LH signaling in the GnRHa group. Furthermore, this may provide an explanation for the benefits of combining GnRHa and hCG (i.e., “dual triggering”) for final maturation of follicles as performed by some clinics.

For FSH, the situation is similar: the modest but physiologically relevant FSH increase induced by GnRHa occurs when *FSHR* expression remains relatively preserved. Therefore, the combination of earlier ligand arrival and higher receptor availability may explain why GnRHa triggers support more physiological follicular maturation and may yield marginally improved oocyte competence. Furthermore, it is noticeable that the recovery of *LHR* expression is higher in the GnRHa group at T = 36h as compared to the hCG-trigger group, which may reflect that LH levels in FF from women receiving GnRHa is declining, while concentrations of hCG remain high in FF from the hCG-trigger group potentially reflecting a stronger ligand induced downregulation of *LHR* expression in GC cells transforming to luteal cells in the hCG group.

The low FF to plasma ratio for FSH for both trigger modalities, the overall low one-digit number concentrations in FF and the rapid downregulation of *FSHR* in GCs may suggest that FSH plays only a minor role in regulating oocyte maturation *in vivo*. However, whether there is an actual effect of the observed peak in FF-FSH at T = 12-17h is unknown. It may be speculated that FSH exerts an effect in the outmost GCs of the follicle and then FSH gradually becomes reduced from the extracellular space as the distance to the antrum is reduced due to binding to the FSHR. At T = 0h *FSHR* expression is already reduced with a level around 1% of levels observed in small antral follicles with a diameter of 2–6 millimeter (23). As FSH is crucial for *in vitro* maturation, it enforces that different hormonal mechanisms are probably involved regulating human oocyte maturation *in vitro* and *in vivo* (24). The successful MII transition of human immature oocytes from small antral follicles depends on the presence of high concentrations of FSH (i.e. 100 IU/L) that probably utilizes the high FSHR expression to secure activation of signaling pathways that induce oocyte maturation, which may not be in operation or at least differently regulated *in vivo* (25).

There were no significant associations between the FSH concentration in circulation at T = 0h and expression of *FSHR* and *LHR* in the corresponding GCs. During OS the concentration of FSH usually reaches a steady state concentration around day 6 (26) with several days to induce LHR expression prior to ovulation induction. This lack of association challenges the current dogma that FSH secures LHR induction in the second half of the follicular phase (27). However, the study of Lindeberg and co-workers found increased *LHR* expression *in vitro* following a stimulation of human GCs with an FSH concentration of more than 250 IU/L approximately a 20 to 30 times higher concentration than observed *in vivo.* We have also observed *LHR* induction in human cumulus cells cultured in the presence of 100 IU/L of FSH (24). However, this may not reflect the *in vivo* conditions and the mechanisms of gonadotropin receptor induction in the second half of the follicular phase in women is still not clarified.

In addition, this study describes the hormonal conditions in FF obtained at T = 0h, which is the most optimal time to evaluate potential differences between effects of different gonadotropin preparations, before the ovulatory trigger induces pronounced differences in the hormonal environment (7, 8). The present study found no difference in *FSHR* and *LHR* gene expression at T = 0h irrespective of which gonadotropin preparation was used for OS (hMG versus rFSH). In contrast, the hormonal conditions in FF at T = 0h were different. The clinical parameters (including age, BMI, total FSH dose administered, and volume of FF at T = 0h) were similar between the two groups and differences are likely to be related to the gonadotropin preparation. Significant differences were observed in the FF concentration of FSH, estradiol and testosterone at T = 0h, while there were interesting trends in the concentrations of inhibin-B and AMH, but the number of observations were probably too low to reach significance. These differences may have clinical implications, and the present results warrants further studies to understand the actions of gonadotropins on follicular development leading up to ovulation.

This study has several limitations. Obviously, the number of observations for each time point is low, and the trigger groups (GnRHa and rhCG treated women) are not completely similar, as the choice of trigger depended on number of follicles > 14 mm before trigger administration (or signs of OHSS), and the stimulation drug (hMG versus rFSH) is not evenly distributed in the trigger groups, which may introduce a selection bias. However, to our knowledge, no other study has documented levels of gonadotropins and gonadotropin receptor expression during the ovulatory process *in vivo*, and the present data provides a first attempt to unravel this physiology including differences between the two modalities of ovulation induction. Further, it would have been an advantage to know the clinical outcome of the oocyte from each of the follicles included, but in most cases an oocyte was not retrieved from the early aspirations (T = 0-32h). The clinical consequences of GnRHa versus hCG trigger in terms of number of mature oocytes - and ultimately pregnancy - cannot be determined by the present study, but it may offer a physiological explanation for results obtained from larger clinical studies indicating a positive effect of GnRHa trigger. As the included cohort represent young women < 36 years, who are relatively FSH sensitive (≥9 large follicles before trigger administration), the uncovered endocrine finding may not apply to all women.

In conclusion, these findings suggest that a GnRHa trigger provides a more physiological, timely, and receptor-optimized stimulus for final oocyte maturation than hCG. For everyday practice, this supports use of agonist triggers in fresh transfer cycles combined with a bolus of hCG (i.e., dual trigger) and agonist trigger alone in freeze-all cycles or in women at risk of ovarian hyper stimulation syndrome to optimize both oocyte quality and luteal safety.

## Data Availability

The original contributions presented in the study are included in the article/**Supplementary Material**. Further inquiries can be directed to the corresponding author/s.
