# Supplementary material for: The ovulation trigger method affects gonadotropin concentrations and gonadotropin receptor expression during final oocyte maturation in women
Source: Front Endocrinol (Lausanne). 2026 Mar 16;17:1791342. doi: 10.3389/fendo.2026.1791342 (PMC13033531; doi:10.3389/fendo.2026.1791342)
Supplement: Supplementary file 1 [file Table1.docx]

**Supplementary table I. Baseline descriptive parameters across time points**

| Parameter | Group 1  0h+36h  (N=23) | Group 2  12h+36h  (N=10) | Group 3  17h+36h (N=6) | Group 4  32h+36h (N=11) | P-value |
| --- | --- | --- | --- | --- | --- |
| Cause of infertility (distribution in numbers) | MF:17, TF:4, UN:0, PCOS:2 | MF:6, TF:0, UN:4, PCOS:0 | MF:2, TF:1, UN:1, PCOS:2 | MF:7, TF:0, UN:2, PCOS:2 | 0.016** |
| Duration between OI and FF collection early puncture (hours) | -11.9 (22,6) | 12.0 (0.2) | 17.0 (0.2) | 32.3 (0.2) | - |
| Duration between OI and FF collection at OPU (hours) | 36.0 (0.1) | 36.0 (0.2) | 36.3 (0.2) | 36.1 (0.2) | - |
| Age (years) | 28.5 (4.9) | 28.0 (4.2) | 28.8 (7.1) | 27.9 (3.5) | NS |
| BMI | 24.8 (5.0)* | 21.8 (3.8) | 21.9 (4.7) | 21.6 (2.4)* | 0.026 |
| Stimulation dosage, total (IU) | 1437 (487) | 1350 (388) | 1275 (1019) | 1200 (418) | NS |
| Number of follicles at last control visit >14 mm | 12 (4) | 11.5 (3.3) | 14 (6) | 14 (6) | NS |
| Size of aspirated follicle T0-T32 (mL) | 3.1 (2.2)* | 3.7 (2.1) | 3.7 (2.7) | 6.8 (3.9)* | 0.001 |
| Size of aspirated follicle T36 (mL) | 4.5 (3.9) | 4.3 (3.1) | 3.6 (3.7) | 5.2 (2.5) | NS |
| S-LH (IU/L) | 4.1 (2.7) | 4.7 (2.3) | 5.6 (2.4) | 5.5 (2.2) | NS |
| S-FSH (IU/L) | 5.2 (2.3) | 6.6 (2.6) | 5.6 (2.9) | 6.5 (2.2) | NS |
| S-Prolactin (mU/L) | 226 (98) | 213 (132) | 191 (106) | 172 (57) | NS |
| S-TSH (mU/L) | 1.7 (0.6) | 1.4 (0.8) | 1.3 (1.5) | 1.8 (1.0) | NS |
| S-SHBG (nmol/L) | 117 (89)* | 166 (94.8) | 132 (28) | 167 (45)* | 0.038 |
| S-Testosterone, free fraction (nmol/L) | 0.019 (0.009) | 0.020 (0.011) | 0.020 (0.004) | 0.015 (0.016) | NS |
| S-DHEAS (µmol/L) | 5.9 (2.3) | 5.7 (3.5) | 5.4 (2.3) | 5.4 (2.4) | NS |
| S-Estradiol (nmol/L) | 0.10 (0.05) | 0.16 (0.08) | 0.12 (0.05) | 0.14 (0.10) | NS |
| S-AMH (pmol/L) | 33.9 (22.7) | 30.3 (29.3) | 28.1 (35.2) | 25.6 (21.2) | NS |

Median (interquartile range) is reported. The differences between the groups were compared by Kruskal-Wallis’ test followed by a post hoc test with Bonferroni correction if a difference was detected. P<0.05 was considered significant. NS = non-significant. *Post hoc test showed a difference between the marked groups. **Fisher’s exact test showed a difference due to a higher number of MF in group 1, whereas no differences were found between the remaining groups. MF: Male factor, TF: tubal factor, UN: Unexplained infertility, PCOS: Non-hyperandrogenic PCOS.

**Supplementary table II. Baseline descriptive parameters across trigger groups**

| **Parameter (Mean (SD))** | **Trigger Drug** | | **p-value** |
| --- | --- | --- | --- |
|  | rHCG (n=17) | GnRHa (n=33) |  |
| **Age at treatment initiation (years)** | 28.5 (5.1) | 27.6 (4.4) | NS |
| **BMI** | 20.9 (2.1) | 23.5 (4.5) | **0.001** |
| **Number of follicles at last control visit >14 mm** | 10 (3) | 14 (4) | **0.006** |
| **S-AMH (pmol/L)** | 25.6 (16.3) | 34.3 (23.9) | **0.033** |
| **S-Estradiol (nmol/L)** | 0.16 (0.1) | 0.11 (0.04) | NS |
| **S-LH (IU/L)** | 5.7 (1.3) | 4.4 (1.9) | NS |
| **S-FSH (IU/L)** | 6.7 (1.7) | 5.3 (1.7) | **0.014** |
| **S-Prolactin (mU/L)** | 201 (175) | 215 (106) | NS |
| **S-TSH (mU/L)** | 1.5 (0.6) | 1.6 (0.8) | NS |
| **S-SHBG (nmol/L)** | 154 (89) | 135 (51) | NS |
| **S-Testosterone free fraction (nmol/L)** | 0.016 (0.009) | 0.0195 (0.01) | NS |
| **S-Androstendion** | 7.4 (5.1) | 6.5 (3.4) | NS |
| **S-DHEAS (µmol/L)** | 5.9 (1.6) | 5.6 (2.7) | NS |
| **Cause of infertility** | MF:9  TF:2  UN:5  PCOS:1 | MF:23  TF:3  UN:2  PCOS:5 | NS* |
| **Stimulation Drug** | rFSH: 17 hMG: 0 | rFSH: 25  hMG: 8 | **0.026*** |
| **Treatment dosage total consummation in IE** | 1350 (325) | 1350 (600) | NS |
|  |  |  |  |

Median (interquartile range) is reported. The differences between the groups were compared by a Mann-Whitney U test. P<0.05 was considered significant.*Fisher’s exact test. MF: Male factor, TF: tubal factor, UN: Unexplained infertility, PCOS: Non-hyperandrogenic PCOS. rFSH: recombinant FSH. hMG: Human menopausal gonadotropin.
